# Supplementary material for: Chemical Composition Analysis, Cytotoxic, Antimicrobial and Antioxidant Activities of Physalis angulata L.: A Comparative Study of Leaves and Fruit
Source: Molecules. 2022 Feb 22;27(5):1480. doi: 10.3390/molecules27051480 (PMC8911865; doi:10.3390/molecules27051480)
Supplement: Supplementary file 1 [file molecules-27-01480-s001.zip › molecules-1592480-supplementary.pdf]

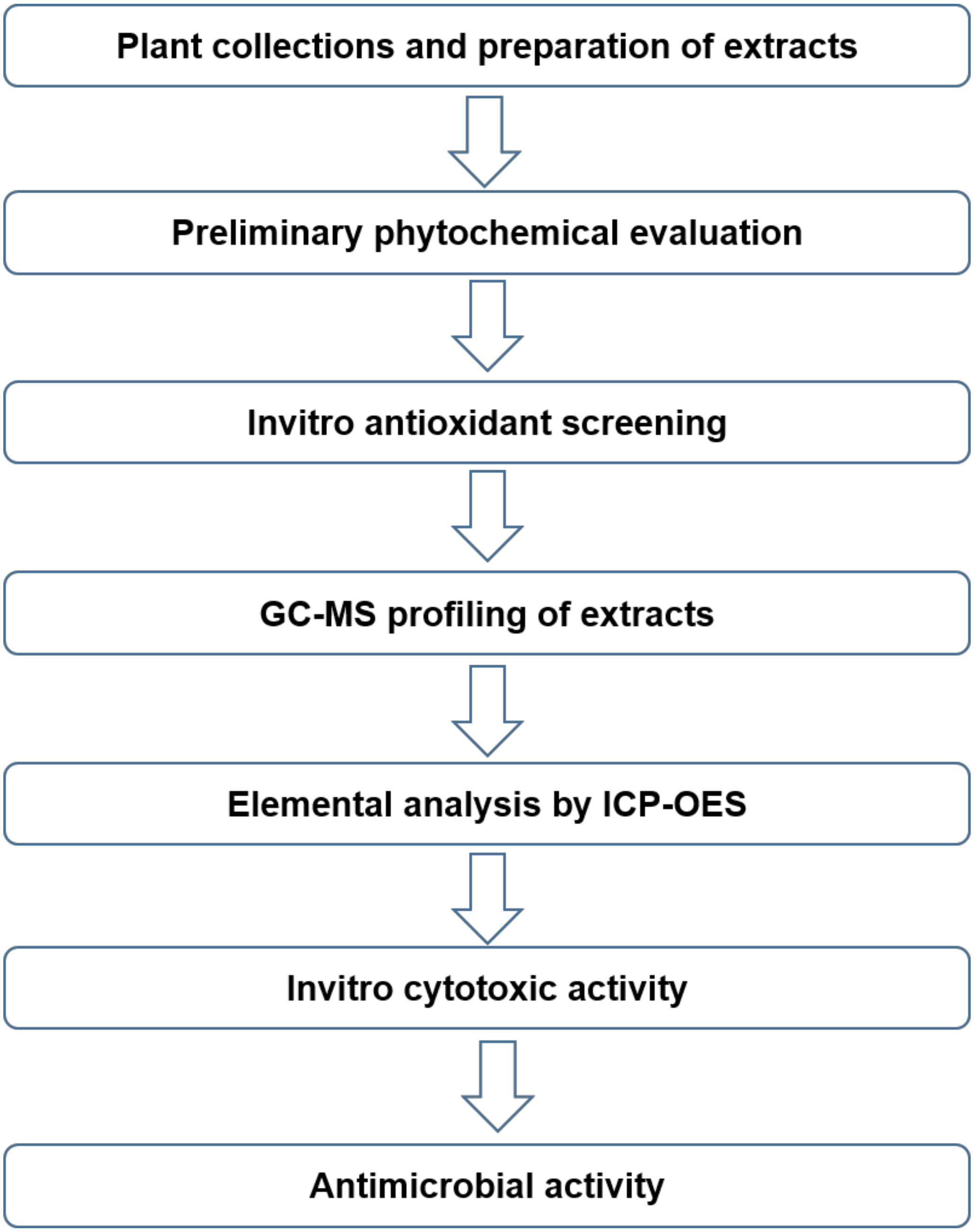

Figure S1. The transparent flowchart of the materials and methods section.

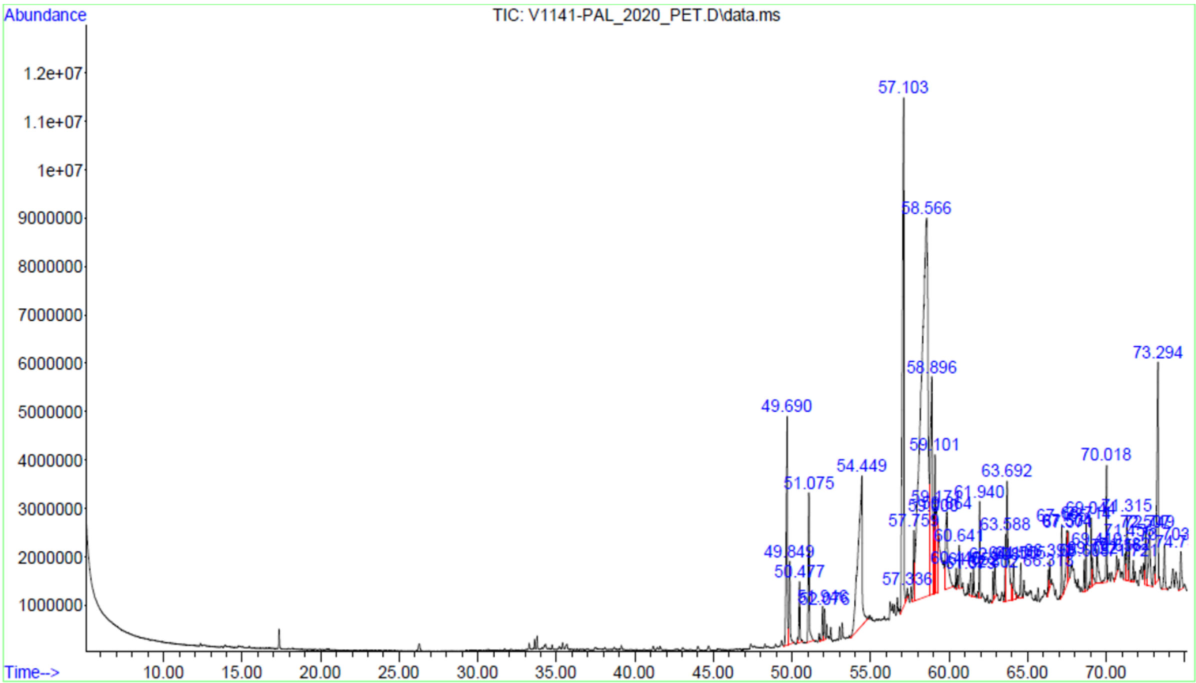

Figure S2. Representative full scan chromatographic profile of ethanolic extract (leaves) of *Physalis angulata*

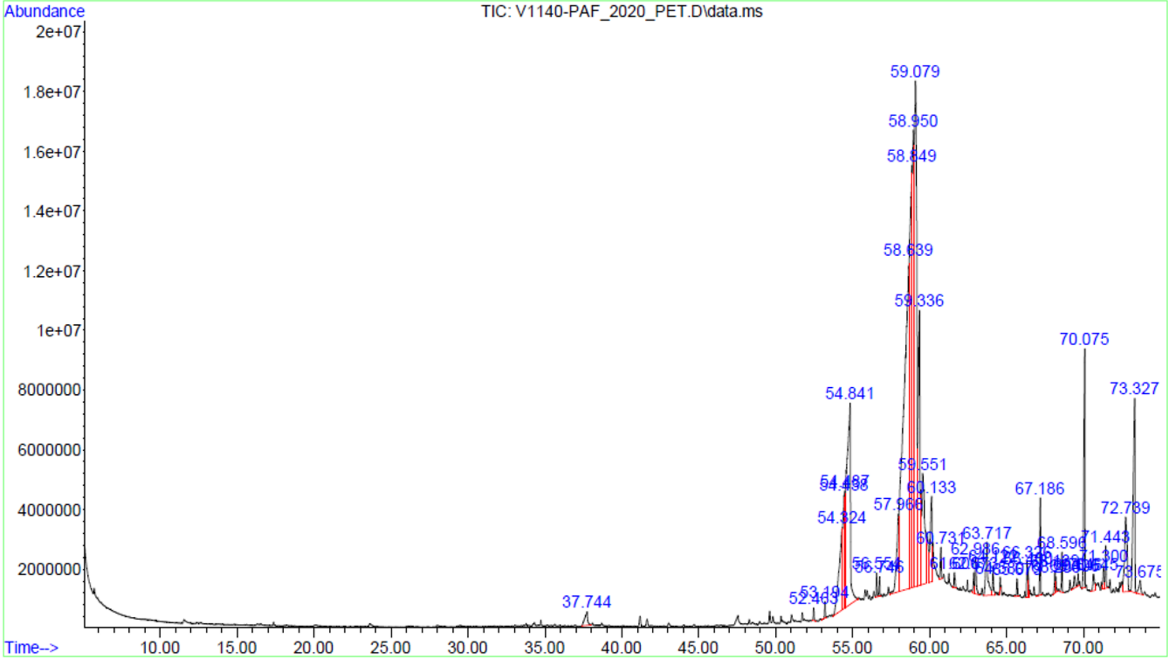

Figure S3. Representative full scan chromatographic profile of ethanolic extract (fruits) of *Physalis angulata.*

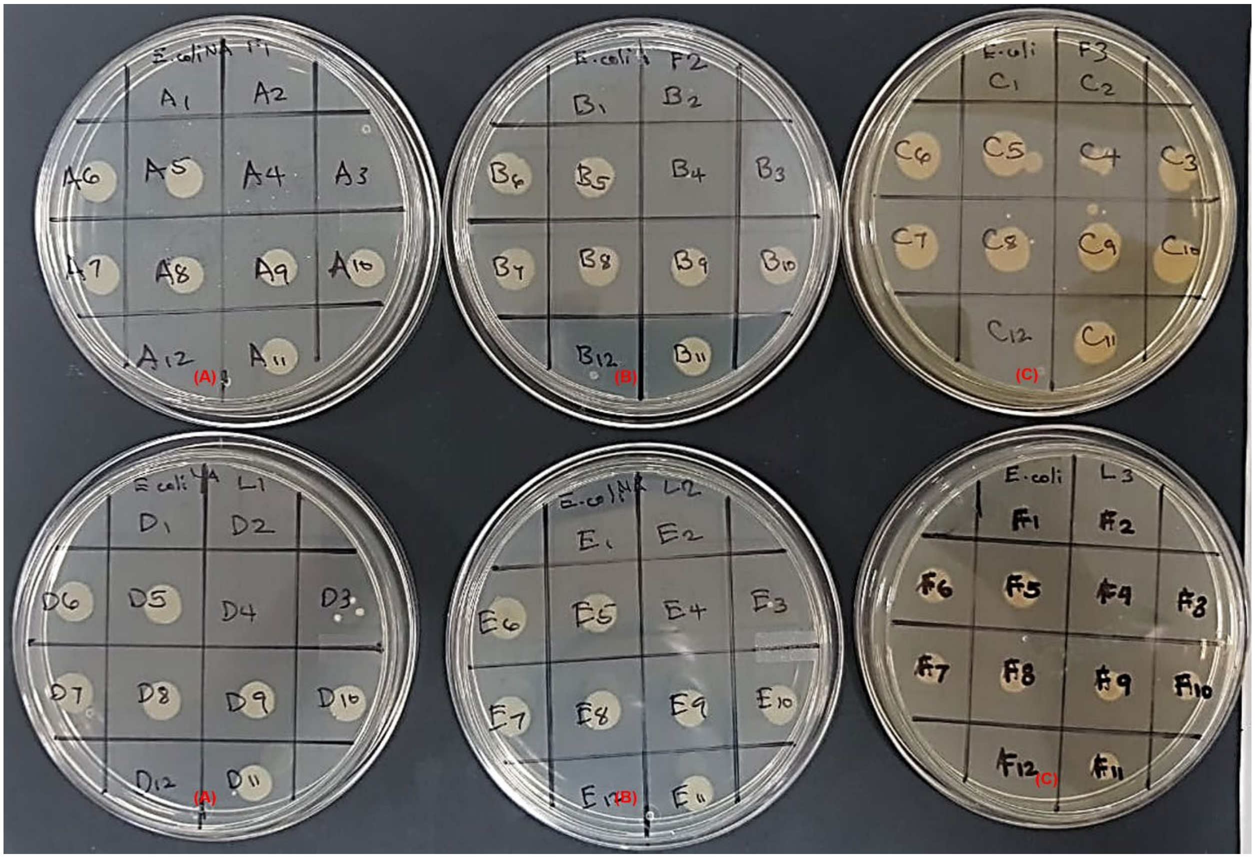

Figure S4. Antibacterial activity of *Physalis angulata* fruit extracts. Plate A: petroleum ether, Plate B: ethyl acetate, and Plate C: ethanol extracts.

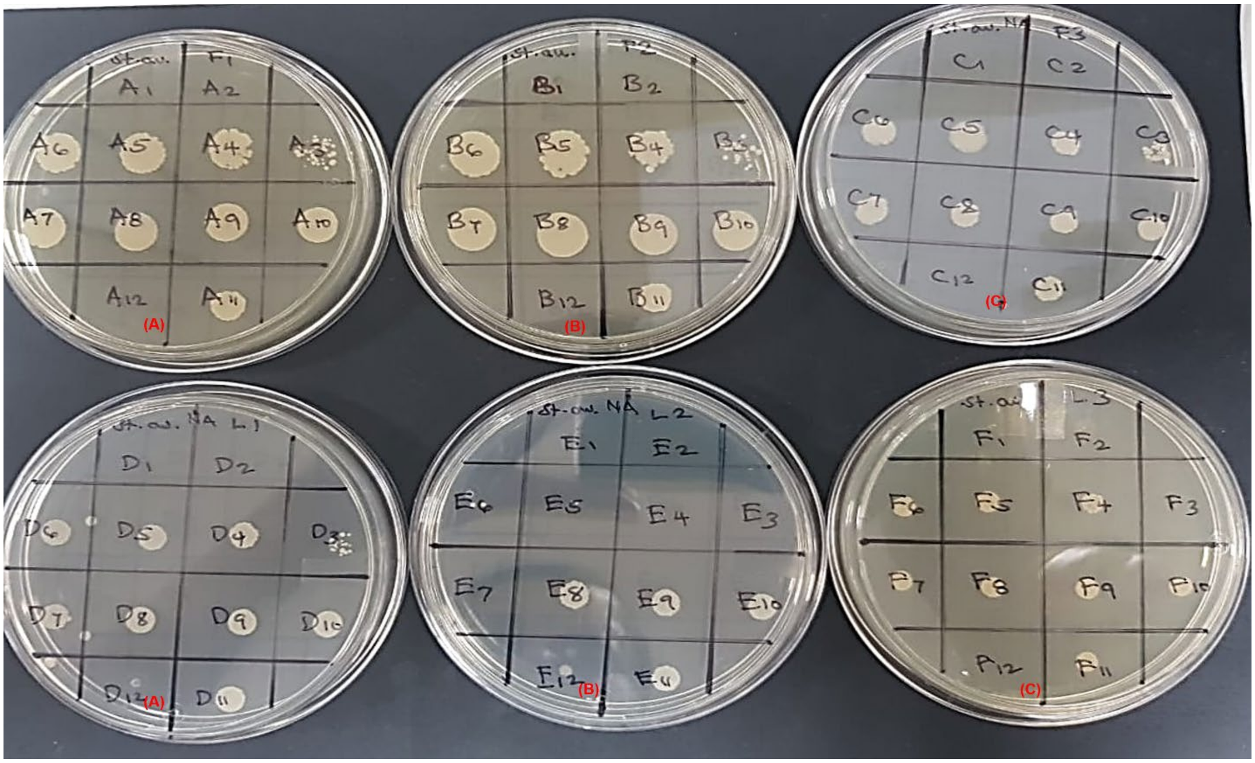

Figure S5. Antibacterial activity of *Physalis angulata* leaves extracts. Plate A: petroleum ether, Plate B: ethyl acetate, and Plate C: ethanol extracts
